# Addressing gender inequality and intimate partner violence as critical barriers to an effective HIV response in sub-Saharan Africa

**DOI:** 10.7448/IAS.17.1.19849

**Published:** 2014-12-11

**Authors:** Charlotte Watts, Janet Seeley

**Affiliations:** Department of Global Health and Development, London School of Hygiene & Tropical Medicine, London, UK

**Keywords:** gender inequality, intimate partner violence, interventions, sub-Saharan Africa

## Abstract

**Introduction:**

In Africa, women and girls represent 57% of people living with HIV, with gender inequality and violence being an important structural determinant of their vulnerability. This commentary draws out lessons for a more effective combination response to the HIV epidemic from three papers recently published in *JIAS*.

**Discussion:**

Hatcher and colleagues present qualitative data from women attending ante-natal clinics in Johannesburg, describing how HIV diagnosis during pregnancy and subsequent partner disclosure are common triggers for violence within relationships. The authors describe the challenges women face in adhering to medication or using services. Kyegombe and colleagues present a secondary analysis of a randomized controlled trial in Uganda of SASA! – a community violence prevention programme. Along with promising community impacts on physical partner violence, significantly lower levels of sexual concurrency, condom use and HIV testing were reported by men in intervention communities. Remme and her colleagues present a systematic review of evidence on the costs and cost-effectiveness of gender-responsive HIV interventions. The review identified an ever-growing evidence base, but a paucity of accompanying economic analyses, making it difficult to assess the costs or value for money of gender-focused programmes.

**Conclusions:**

There is a need to continue to accumulate evidence on the effectiveness and costs of different approaches to addressing gender inequality and violence as part of a combination HIV response. A clearer HIV-specific and broader synergistic vision of financing and programming needs to be developed, to ensure that the potential synergies between HIV-specific and broader gender-focused development investments can be used to best effect to address vulnerability of women and girls to both violence and HIV.

## Introduction

Gender inequality and violence against women remain important drivers of the HIV epidemic, particularly in sub-Saharan Africa, where women and girls represent 57% of people living with HIV [1]. It has long been recognized that gender inequality is a core issue that underpins women's vulnerability to HIV in the region, with a combination of social, economic and cultural factors contributing to the higher levels of HIV infection occurring among women [2].

What is emerging also is a recognition of the scale of violence against women, and its multiple connections with HIV. Estimates, published in *Science* last year, suggest that globally 30% of partnered women will experience physical or sexual violence from a partner, with regional figures for sub-Saharan Africa of 37% [3]. The gender-related aspects of HIV stigma, including that violence and the fear of violence may act as important barriers to women accessing services, testing and disclosure, have long been documented. In a previous systematic review published by JIAS, longitudinal epidemiological evidence also shows that women who have a history of partner violence are at an increased risk of incident HIV infection, with a meta-analysis of the three existing cohort studies finding incidence rate ratios of 1.22 (1.0–1.64) [4]. The growing body of evidence on the scale and consequences of gender inequality and violence has helped garner greater international attention to the issue, with strong arguments for the inclusion of an indicator on violence against women to be included in the post-MDG sustainable development agenda. This commentary discusses and draws out some of the over-arching lessons from three papers [5,6,8], recently published in JIAS, that provide greater insights into the ways in which violence against women may undermine the current HIV response; provide hope that it is possible for programmes to address gender inequality and violence; and set out some of the shortcomings in the current evidence base.

## Discussion

Hatcher and colleagues [5] use the findings from qualitative data collected from women attending ante-natal clinics in Johannesburg to discuss the complex relationship between gender inequality, violence and HIV in pregnancy, and the implications for prevention of mother to child transmission (PMTCT) programmes. Respondents described how HIV diagnosis during pregnancy, and subsequent partner disclosure, are common triggers for violence within their relationships, with their disclosure of infection causing conflict, usually related to perceived infidelity and the notion that women are “bringing” the disease into the relationship. They also describe how adherence to medications or service use could be difficult, as women feared that by doing this they may alert their partners to their HIV status. Pregnant women also described how they felt unable to refuse sex or negotiate condom use – prioritizing their and their unborn baby's physical safety during pregnancy over the issue of potential secondary HIV transmission. This research illustrates how, even with a highly effective prevention technology, such as PMTCT medication, violence and the fear of violence pose an important barrier to the elimination of vertical HIV transmission, and to ensuring that broader maternal and child health goals are met.

These challenges highlight the need for programmes that rely on HIV testing to acknowledge and deal with the realities of power inequalities and violence in relationships. Although this may seem overwhelming as a goal, Kyegombe *et al*. [6] present data from the secondary analysis of the SASA! trial: the first randomized controlled trial in sub-Saharan Africa to assess the community level impact of a community mobilization intervention that seeks to prevent violence against women. SASA! supports male and female community activists and stakeholders to support a process of critical reflection about power. A range of formal and informal approaches are used to support discussion about how men and women may use power negatively, but also how people can use their power to promote greater equality between men and women, and reduce violence and HIV. The findings on the impact of the intervention suggest that the levels of violence were 52% lower in intervention communities, although the findings were not statistically significant [7]. Kyegombe and colleagues paper explores whether SASA! also had an impact on HIV-related attitudes and relationship dynamics. Interestingly, men in intervention communities reported adopting a broad range of HIV protective behaviours in the past year, including significantly lower levels of sexual concurrency, and higher reported levels of condom use and HIV testing than men in the control communities. Men were also more likely to report increased joint decision-making, open communication, and appreciation of their partner's roles. Women had similar responses, although with smaller differences between intervention and control communities. Qualitative data collected suggest that the intervention led to a changing dynamic within relationships, including increased trust, co-operation and communication, with the intervention helping men and women to work more effectively together to resolve problems.

SASA! is not the only promising intervention to address gender-relations and HIV. Remme *et al*. [8] present a systematic review of current evidence on the costs and cost-effectiveness of gender-responsive HIV interventions. Encouragingly, the review identified a broad range of interventions that have been shown to have a promising impact either on HIV or its proximate determinants. Interventions identified included sex worker collectivization and peer support empowerment strategies; interventions providing education, skills training and supporting the development of women's self-efficacy; multi-media approaches that challenge the acceptability of violence and promote HIV behaviour change; group-based participatory training involving men, boys, women, girls; and economic support to poor women, female sex workers and schoolgirls. However, despite this ever-growing evidence base, most interventions had no economic analyses, making it difficult to assess their costs or value for money in comparison to other potential areas of investment. The paucity of the cost evidence base points to a large research gap, given the need to identify cost-effective and affordable responses to the HIV epidemic.

## Conclusions

Moving forward, these papers illustrate the potential for programming on gender and violence along the prevention – treatment spectrum of the HIV response. This is not a luxury but a necessity for effective programming, especially in settings where there is a high HIV prevalence among women. The above three papers also serve as a timely reminder of the need to actively engage with both men and women in the implementation of gender-related programmes, and to address the particular vulnerabilities, social pressures and economic challenges that men and women face, which commonly drive violence and HIV risk behaviours. Although the evidence base is increasing, there is a clear need for further data on what approaches to programming are most effective, and for greater attention to be paid to learning about the costs of delivering such interventions at scale.

Resources provided for HIV interventions cannot solve the issue of gender inequality and violence alone. Investment in broader social development and interventions which directly address gender inequalities, are also needed. A useful way to map out options was used by Remme and colleagues in their paper [8], and relates to the HIV investment framework [9]. For this, interventions were grouped into three types: gender-responsive activities that can be added to basic HIV programmes to enhance their effectiveness and efficiency by addressing gender-related barriers to behaviour change, service uptake and retention (*HIV*+); HIV-specific interventions that could be added onto gender-responsive development programmes, to achieve a synergistic HIV effect (*Gender*+); and gender-responsive development interventions that do not explicitly include programmatic HIV components, but may nevertheless have secondary HIV-related benefits due to their impact on gender inequalities and/or violence (*Gender*). Using this framework to take as an example related to Hatcher and colleagues’ paper, described above, a health systems intervention that seeks to identify and support women attending Antenatal care (ANC) services who are experiencing violence would fall within HIV+, and should be considered for funding from HIV resources, as a critical enabler to effective PMTCT programming. While, community transformative interventions, such as SASA!, described by Kyegombe *et al*. [6], could potentially fall within the broader gender investment focus, meriting co-financing across gender and HIV budgets.

This thinking of core and co-investment strategies could be further developed, as discussions around the future financing of the HIV response, and the broader vision of the post-MDG agenda evolve. This could be used to gain a clearer HIV-specific and synergistic vision of programming to be developed, alongside the continued accumulation of evidence on what approaches to gender-responsive combination programming may be most effective.

## References

[1] UNAIDS (2012). Report on the global AIDS epidemic.

[2] UNAIDS Agenda for accelerated country action for women, girls, gender equality and HIV. http://www.unaids.org/sites/default/files/media_asset/20100226_jc1794_agenda_for_accelerated_country_action_en_0.pdf.

[3] Devries KM, Mak JY, García-Moreno C, Petzold M, Child JC, Falder G (2013). Global health. The global prevalence of intimate partner violence against women. Science.

[4] Li Y, Marshall CM, Rees HC, Nunez A, Ezeanolue EE, Ehiri JE (2014). Intimate partner violence and HIV infection among women: a systematic review and meta-analysis. J Int AIDS Soc.

[5] Hatcher AM, Woollett N, Pallitto CC, Mokoatle K, Stöckl H, MacPhail C (2014). Bidirectional links between HIV and intimate partner violence in pregnancy: implications for prevention of mother-to-child transmission. J Int AIDS Soc.

[6] Kyegombe N, Abramsky T, Starmann E, Devries KM, Michau L, Nakuti J (2014). The impact of SASA!, a community mobilisation intervention, on reported HIV-related risk behaviours and relationship dynamics in Kampala, Uganda. J Int AIDS Soc.

[7] Abramsky T, Devries K, Kiss L, Nakuti J, Kyegombe N, Starmann E (2014). Findings from the SASA! Study: a cluster randomized controlled trial to assess the impact of a community mobilization intervention to prevent violence against women and reduce HIV risk in Kampala, Uganda. BMC Medicine.

[8] Remme M, Siapka M, Vassall A, Heise L, Jacobi J, Ahumada C (2014). The cost and cost-effectiveness of gender-focused interventions for HIV: a systematic review. J Int AIDS Soc.

[9] Schwartlander B, Stover J, Hallett T, Atun R, Avila C, Gouws E (2011). Towards an improved investment approach for an effective response to HIV/AIDS. Lancet.

